# Elongational Stresses and Cells

**DOI:** 10.3390/cells10092352

**Published:** 2021-09-08

**Authors:** Kylie M. Foster, Dimitrios V. Papavassiliou, Edgar A. O’Rear

**Affiliations:** Department of Chemical, Biological and Materials Engineering, University of Oklahoma, Norman, OK 73019, USA; kylie.m.foster-1@ou.edu (K.M.F.); dvpapava@ou.edu (D.V.P.)

**Keywords:** elongational stress, elongational flow, cell mechanics, microfluidics, hemolysis, cell damage

## Abstract

Fluid forces and their effects on cells have been researched for quite some time, especially in the realm of biology and medicine. Shear forces have been the primary emphasis, often attributed as being the main source of cell deformation/damage in devices like prosthetic heart valves and artificial organs. Less well understood and studied are extensional stresses which are often found in such devices, in bioreactors, and in normal blood circulation. Several microfluidic channels utilizing hyperbolic, abrupt, or tapered constrictions and cross-flow geometries, have been used to isolate the effects of extensional flow. Under such flow cell deformations, erythrocytes, leukocytes, and a variety of other cell types have been examined. Results suggest that extensional stresses cause larger deformation than shear stresses of the same magnitude. This has further implications in assessing cell injury from mechanical forces in artificial organs and bioreactors. The cells’ greater sensitivity to extensional stress has found utility in mechanophenotyping devices, which have been successfully used to identify pathologies that affect cell deformability. Further application outside of biology includes disrupting cells for increased food product stability and harvesting macromolecules for biofuel. The effects of extensional stresses on cells remains an area meriting further study.

## 1. Introduction

Fluid forces and shear stress have become widely appreciated in biology and medicine for their effects on cells. Flow stimulates the release of the potent vasodilator nitric oxide (NO) in the circulatory system [1,2] and affects anatomy and differentiation in developmental biology [3,4]. The potential harmful effects of stresses on the cellular and molecular components of blood must be considered when engineers design hemodialysis units and blood-contacting medical devices [5,6,7]. Both the food and biotechnology industries depend on knowledge of cell mechanics, and the effects of stresses, for optimal processing conditions for products. An appreciation of forces acting on cells is important to understanding many fundamental mechanisms occurring at the cellular scale as well as the response of cells to forces in applied settings.

**Figure 1:**
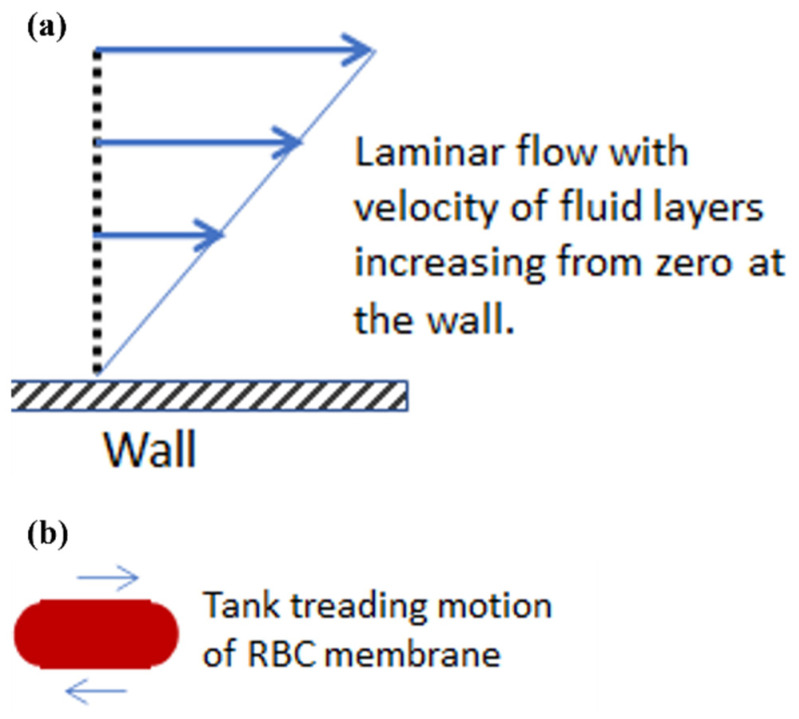
Shear stresses and their effect on red blood cells in laminar flow. (**a**) Velocity profile of a fluid flowing past a wall in laminar flow. (**b**) Red blood cell exhibiting a tank treading motion due to shear stresses.

Shear stress corresponds to just one of the types of forces present during flow. Its mode of action is different than that of an extensional stress. Frictional in nature, shear stresses act at the wall during flow in a tube or blood vessel to oppose the driving force due to pressure. Shear stress or friction also exists between layers of fluid traveling at different velocities in what is called laminar flow, represented in Figure 1a. The friction at the wall is such that the fluid layer in immediate contact with the wall has zero velocity. Away from the wall, velocity increases and faster layers apply shear stress on adjacent slower layers in an effort to increase their speed. At the same time, slower layers try to hold the faster layers back with a force opposite to the direction of flow as the effects of friction propagate through the fluid. It has been found experimentally that the very flexible red blood cell (RBC), when placed in a shear stress environment, undergoes an antisymmetric motion called “tank treading”, shown in Figure 1b [8]. In this case, the cell membrane rotates about the interior of the red blood cell. This is in contrast to other cell types whose rotation in shear flow is more like a solid body. In the circulatory system, shear forces also support leukocyte rolling and adhesion to the endothelium of the vasculature as leukocytes proceed to move out into the tissue to fight infection or contribute to wound healing [9].

**Figure 2:**
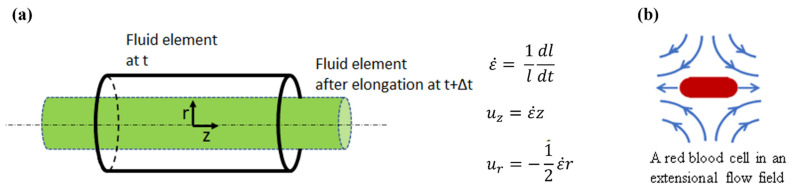
Elongational flow and its effects on red blood cells, as a representative example of cells in general. (**a**) Uniaxial elongational flow proceeds with a lengthening at the ends and contraction at the center. In a system of set geometry, the elongation rate $\dot{\varepsilon }$ at some point in the flow field will increase with applied flow rate. (**b**) Red blood cell deforming under elongational flow with no membrane rotation.

Less well known and understood are the effects of extensional or elongational stresses that are usually present along with shear stresses in many biological flow systems. Typical cases of extensional stresses are found in sudden contractions or expansions of the flow field. For example, many medical devices induce extensional stresses that often remain unaccounted including the entrance and exits of ventricular assist devices, reverse gap flow in artificial heart valves, hypodermic needles, and others [10]. Stresses that are elongational in nature also aid the processing of foods (e.g., tomato concentrates) and biopharmaceuticals (e.g., proteins expressed in *E. coli*) [11,12]. Considering the prevalence of elongational stresses in many flow systems, it is important to understand how they affect cells and how they compare to shear stresses. In uniaxial elongational flow, a fluid element is stretched at a constant strain rate $\dot{\varepsilon }$ so that it narrows (Figure 2a). A constant strain rate means that the distance *l* between two adjacent points grows at an exponential rate for a simple fluid and, as such, cell exposures to elongational flow tend to be brief and intermittent due to acceleration of the cell in the flow field. In contrast to the tank tread motion of a red cell undergoing shear, a symmetric stretching of the cell in an extensional flow field does not result in membrane motion (Figure 2b). For both shear and elongational stress, however, the aspect ratio of the cell increases until it will not deform further, or the membrane fails. The aspect ratio is defined as the ratio of the length to the width for the projected area of the cell, observed in the video microscopy recordings.

Deformation of fluids has been studied for a long time due to its importance in the polymer and textile industries [13]. Rheological studies based on both experiments and theory have contributed to the development of commercial processes including polymer processing, fiber spinning, and filament stretching. The effects of shear stresses on cell mechanics have long been studied, often neglecting any extensional stresses that may be present—for example, a recent article reviewing 100 years of extensional flows does not mention applications to cells, though there has been some early work on biomolecules [14]. Extensional stress experiments have been carried out on cervical macromolecules for the detection of ovulation [15]. More recently, elongational flow has been a valuable tool in validating experimental results for computational modeling of macromolecules like DNA [16]. Perkins et al. examined individual molecules of fluorescently labeled DNA in an extensional flow to learn about polymer dynamics [17] while several recent studies have examined unfolding of the von Willebrand Factor (vWF) and flow-induced aggregation [18,19,20]. 

In this review article, we look at methods used to study cells in an extensional flow field and the effects of elongational stresses on cells. The motivation to explore cell rheology derives in part from injury to the cell which, depending on the circumstances, can be either beneficial or detrimental. In addition, rheological studies have been used to characterize cell phenotype, cell age, cell pathologies, and cell activation. Recent work, discussed below, has shown that extensional stresses have significant effects on cells, and thus must be considered in order to have a more comprehensive understanding of cell rheology. 

## 2. Experimental Methods for Extensional Stresses on Cells

Several well-known methods have been established to examine the effect of shear stresses on cells. These include viscometers used to apply uniform levels of shear stress to suspensions and rectangular microfluidic channels [21,22,23]. Devices used for observing cells under extensional flow also exist with several options for the bioscientist or bioengineer wanting to study the effects of elongational flow on cells, as will be discussed below and summarized in Table 1. These fluidic devices typically have a small region where pure extensional flow exists. 

### 2.1. Microfluidic Systems Based on Flow through a Contraction

Microfluidic devices utilizing bilaterally symmetric constrictions have been used to create extensional flow regions. In general, flows in microfluidic devices exhibit low Reynolds numbers resulting in laminar flow and parabolic velocity profiles. Away from the center of the parabolic profile shear stresses are present, whereas the middle of the profile is a shear free zone providing a region of pure elongational stresses when flowing through a constriction. These devices are typically prepared using soft microlithography which places practical limits on the dimensions of the channel, particularly in the depth, and thus on the size of cells suitable for study.

**Figure 3:**
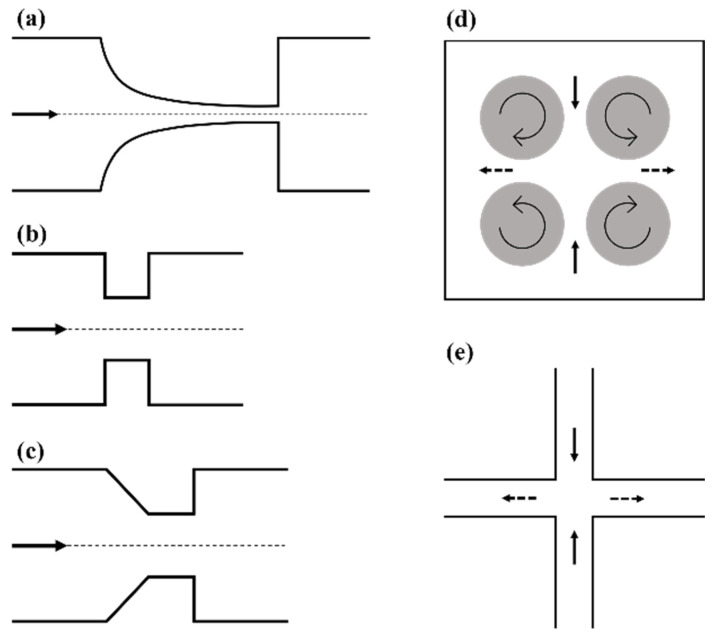
Representative schematics of various extensional flow devices. (**a**) Hyperbolic converging channel, (**b**) abrupt constriction, (**c**) tapered constriction, (**d**) Taylor’s four-roll mill, and (**e**) cross-flow channel. All arrows show the direction of flow. In (**d**,**e**) solid arrows denote the compressional flow axis and dashed arrows denote the extensional flow axis.

Constrictions following a hyperbolic curve are the ideal option and have been commonly used [24,25,30,31,32,33,34,35]. The basic design of a hyperbolic microfluidic device can be seen in Figure 3a, where the hyperbolic curvature of the vessel walls results in the creation of a region of pure extensional flow along the centerline of the channel, free from any shear stress. This unique geometry results in a cross-sectional area that is inversely related to the axial position, which results in a linear increase in velocity along the centerline [24,34]. This linear increase in velocity results in a constant elongational rate along the center line, as expected from the xx component of the extensional rate for inviscid flow

(1) $${\dot{\varepsilon }}_{xx}=\frac{\delta u_{x}}{\delta x}$$

and the apparent extensional rate, assuming plug flow (i.e., neglecting any shear flow from the wall), can be estimated according to

(2) $$\dot{\varepsilon }=\frac{Q}{L_{c}d}\;\left(\frac{1}{w_{d}}-\frac{1}{w_{u}}\right)$$

where *Q* is the volumetric flow rate through the channel, $w_{u}$ and $w_{d}$ are the initial and final widths of the contraction, respectively, *L_c_* is the length of the contraction, and *d* is the depth of the channel [24,25,31,34,36]. This equation is strictly only valid when shear effects can be neglected, which is true along the centerline in channel flows. The magnitude of the extension rate is easily controlled, for a given geometry, by adjusting the volumetric flow rate. Ober et al. reported that flow velocimetry measurements of Newtonian fluids show a spatially uniform extension rate as expected, but the value predicted by equation (2) was 66% lower than the true extension rate [24]. They explained that this discrepancy is the result of a 2D approximation of a 3D flow as well as the geometric abruptness of the contraction and the non-rectangular cross-section of the duct due to their fabrication process. To increase the magnitude of the stress in these systems, polymers are often added which increases the viscosity, but may also make the media viscoelastic. Viscoelasticity of the fluid is often denoted by the Deborah number (De), a dimensionless number reflecting the ratio of the elastic forces to the viscous forces or, basically, the relative solid and liquid characters of the material. Higher De numbers signify increased viscoelasticity, which can have significant effects on the flow in the hyperbolic channels. Fluids with low Deborah numbers (De < *O*(1)) behave similar to a Newtonian fluid, as predicted above. For De > *O*(1) upstream vortices develop that do not affect the spatially homogenous extension rate, while De > *O*(10) results in a flow that has temporal variations making the determination of the extension rate unreliable [24]. Other limitations to the device are more so related to device construction. Leaking of the channels at high flow rates is not uncommon and limits the achievable maximum extensional rates. In the case of Lee et al., they achieved a maximum rate of 56.2 s^−1^ for a flow rate of 100 µL/min, achieving a maximum extensional stress of around 3 Pa, while higher flow rates resulted in leaking [31]. Faghih et al. overcame this limitation and have produced channels withstanding pressures of 350 Pa without leaking, achieving much larger extensional stresses of around 100 Pa [25]. One last limitation is the need to focus the trajectories of particles of interest into the area of pure extensional flow. Cells that are away from the centerline experience levels of shear stress that increase as they get closer to the channel walls. Piergiovanni et al. sought to address this by constructing their microfluidic device with two side channels that delivered sheath fluid to focus their cells along the centerline [34], while Faghih et al. chose to limit observations to a region in a central band of 20 µm [25]. When it comes to measuring cellular deformation, cell focusing increases the cells found near the centerline of the channel and thereby increases the number of observable cells over a given time period. Focusing the cells along the centerline would be more important when attempting to quantify the presence or absence of cell markers such as lactate dehydrogenase (LDH) release. The lack of trajectory focusing would cause contribution of shear stresses to the response.

In addition to hyperbolic contractions, abrupt and tapered constrictions have also been used to study extensional flows [10,26,37,38]. Their basic design can be seen in Figure 3b,c. As with hyperbolic constrictions, the extensional stresses are only found near and within the contraction region due to the increase in velocity experienced there. Computer simulations for abrupt constrictions show that the extensional flow region begins approximately one orifice diameter before the constriction, and that the maximum extension rate is experienced just before the constriction entrance [37]. Similar to hyperbolic channels, pure extensional flow is only experienced along the centerline of the channel, due to the zero shear stress there, but unlike in hyperbolic channels the extension rate is not constant. This is due to a non-linear increase in velocity through the contraction, thereby resulting in a non-constant extension rate, as seen in Equation (2). Computational fluid dynamics (CFD) analysis of both abrupt and tapered constrictions by Yen et al. showed that the maximum extensional stresses were found at the corners of both types of contractions and that this was also an area of high shear stress as expected [10]. As previously mentioned, there is a region of pure extensional flow along the centerline of the contraction, but the magnitude is smaller than that experienced at the corners [10]. CFD also showed that abrupt contractions result in larger extensional stresses than tapered contractions [10]. Although the extension rate is not constant, it is easily controlled by altering the flow rate, as with the hyperbolic channels. Using a tapered contraction, Mancuso et al. reported a linear increase in maximum extension rate with increasing volumetric flow rate and observed an extension rate of 2000 s^−1^ at a flow rate of 5 µL/min [26]. Aside from the non-constant extensional rate and regions of combined extensional and shear stresses, other disadvantages also exist for tapered and abrupt channels. With both, upstream vortices and transient instabilities develop with increasing flow rates and increasing viscoelastic character. As with hyperbolic constrictions, focusing the cells along the centerline would also be beneficial to decrease the effects of shear in bulk measurements as well as increase the number of observable cells in cell deformation studies.

### 2.2. Taylor’s Four-Roll Mill

Seldom used today, Taylor’s four-roll mill was one of the earliest reported devices used to create an extensional flow field. The four-roll mill was developed by Taylor to study the breakup of liquid droplets, and was a simple device that created a two dimensional, pure extensional flow [39]. The device as seen in Figure 3d is made up of four identical cylinders, each centered such that they sit on the corners of a square, all typically housed in a square box. The cylinders rotate at a given angular velocity with the neighboring cylinders rotating in opposite directions creating two opposed laminar streams. The streamlines of this flow field are rectangular hyperbolas [28,40]. This creates an extensional flow field, with a uniform extension rate, in the center of the area confined by the four cylinders as well as a stagnation point, defined as having zero velocity. Typically, the object of interest would be trapped and observed in the stagnation point where both attractive and repulsive forces are experienced along the compressional and extensional axes, respectively [28]. The extension rate can be predicted using

(3) $$\dot{\varepsilon }=2\pi \Omega \kappa$$

where Ω is the angular speed of the rollers, and *κ* is a proportionality constant that varies with the fluid medium and design parameters of the mill [28]. Several groups have characterized the flow in the four-roll mill and studied its limitations [40,41,42]. One of the first limitations being the difficulty of maintaining the object of interest in the stagnation point, as reported by Taylor, which can be addressed by adjusting the speed of the left or right pair of cylinders [39]. Additionally, it has been found that with increasing Reynolds numbers, pure extensional flow becomes unstable, developing counter rotating vortices aligned in the stretching direction [42] and a loss of symmetry in the flow pattern [41]. Flows below the critical Reynolds number ($Re_{\gamma ,cr}\sim 17$) are stable [42]. The low critical Reynolds number limits the achievable extensional rates in the four-roll mill, not to mention that its size is not suited for examining individual cells. Akbaridoust et al. did construct a functioning miniature four-roll mill suitable for studying cells, but not without difficulties [28]. Their miniature four-roll mill had fluctuations in the stagnant point position up to 50 µm, due to eccentric rotation of the rollers, and was only able to achieve a maximum strain rate of 6 s^−1^ in a 99% glycerol solution. The already small size of the device made it difficult and expensive to address these issues.

### 2.3. Cross-Flow Microfluidic Systems

Cross-flow microfluidic devices were a natural progression from Taylor’s four-roll mill, and resolved the limitations of Akbaridoust’s miniature four-roll mill. The cross-slot microchannel, seen in Figure 3e, creates a very similar flow field. The device consists of two opposing inlets channels perpendicular to two opposing outlet channels. Fluid is injected into the inlets and can either be withdrawn at a defined rate or simply allowed to exit though the outlets. This bounded flow provides better flow stability allowing higher strain rates to be achieved, providing an advantage over the four-roll mill [43]. The device creates a planar extensional flow similar to Taylor’s four-roll mill with a similar velocity field, hyperbolic streamlines, and a stagnation point in the center where pure planar extensional flow is experienced [28,44]. Objects are trapped in the stagnation point for a finite amount of time and experience compression along the inlet axis and extension along the outlet axis as in the four-roll mill [44,45]. The extensional rate at the stagnant point is inversely related to the channel dimensions according to

(4) $$\dot{\varepsilon }\approx \frac{2U}{w}$$

where *U* is the average flow velocity in the inlet/outlet channels and *w* is the channel width [43,46]. Theoretically, this device can achieve high extensional rates, easily controlled by adjusting the volumetric flow rates. Akbaridoust et al. achieved strain rates up to 142 s^−1^ in their cross slot microchannel, which was significantly greater than that achieved in their miniature four-roll mill [28]. Similar to the different types of constriction devices discussed above, cross-slot channels only have a small region of pure extensional flow with shear stresses existing to varying degrees outside of this area. Therefore, it is important to determine the area of uniform extension rate, where cell deformation can be observed. The geometries of cross-slot channels are often described by using a dimensionless number (α), where α is the ratio of the channel depth (d) and width (w). Two-dimensional (2D) numerical simulations of a cross-slot channel with an infinite α show that in a radius of w/16 from the stagnation point the extension rate changes less than 5% [44]. Flow velocimetry measurements show an even larger radius (w/4) of uniform extension rate in channels with an α of 0.53 [47]. Microparticle image velocimetry (micro-PIV) measurements on a channel with α = 0.1 showed that a central region of 0.6 w × 0.6 w resulted in an extension rate variation of 2%, as well as stagnation point variations limited to 1 µm [28]. Clearly, the area of uniform extension rate depends on the channel dimensions and on the flow rates. While these 2D simulations are advantageous, it should be kept in mind that the flow is never truly 2D, and the boundaries on the top and bottom of the device will have effects through the depth of the channel, even for larger values of α which do ensure a more uniform extension rate through the z-axis [43]. As with all the devices discussed thus far, at high enough flow rates or with highly viscoelastic fluids, instabilities occur along with asymmetric flows fields. The asymmetric flow field, characterized as a forward bifurcation, was also confirmed by numerical simulations [48]. Lower values of α had a stabilizing effect for these instabilities [43], but this must be balanced with the fact that a larger α better approaches a 2D planar extensional flow field. Another limitation of cross-slot microchannels is that the cell trajectory through the channel has significant effects on the strain rates experienced and thus the extent of deformation measured. Henon et al. performed numerical simulations of RBCs flowing through cross-slot microchannels and found three deformation modes depending on the entry position [27]. The first occurs in cells flowing along the centerline of the inlets, which experience very little shear stress and upon reaching the center, near the stagnation point, show large deformations along one axis and remain largely symmetrical. Cells in between the centerline and the walls make up the second mode of deformation, experiencing limited levels of shear stress in the channel arms prior to extensional stresses in the center of the channel leading to asymmetric deformations. The last deformation mode occurs for cells traveling near the wall that experience the highest shear in the channel arms as well as additional shear in the area located between the channel center and the corners resulting from boundary effects, this results in highly asymmetrical cell deformation. Limiting observations to cells in the first category or unifying the cell trajectories, providing a more uniform kinematic history, would yield more accurate deformability measurements. Several studies have used inertial or viscoelastic focusing in cross-slot microchannels to achieve limited cell trajectories [45,49,50]. Using inertial flow (Re ~*O*10^−1^) to focus cell trajectories the kinematics of the flow field significantly changed compared to an inertia-less field [45]. Under inertial flow, particles decelerated closer to the stagnation point and had dramatically changed streamlines in the flow field [45]. The strain rate gradient gradually increased with increasing Re, thus shrinking the region with uniform pure extensional flow, and at Re ≥ 40 vortices developed near the curved walls where the channel arms intersected (i.e., the intersection had rounded corners) [45]. Viscoelastic focusing results in an almost identical flow field to the inertia-less Newtonian case, producing similar velocity fields, streamlines, and regions of uniform strain [45]. Although, using a viscoelastic focusing method, one must consider the issues discussed above for highly viscoelastic fluids. Using such cell focusing methods would produce more accurate measurements of cell deformability and more accurate bulk measurements of cellular damage.

### 2.4. Optical Tweezers

Optical tweezers can also be used to expose cells to forces that would cause similar deformations experienced under elongational flow. The basis of this method is the ability to trap an object, commonly a silica bead, in a laser beam. This is possible because as the photons from the laser pass through the object they undergo a change in momentum exerting a force which pushes the object to the laser’s focal point, thus trapping the object [29]. By attaching beads to the surface of cells, the beads can then be trapped in the optical tweezers and moved, thus stretching the cell. This method allows for easy control and manipulation of the forces exerted onto the cell but is limited as only one cell can be manipulated at a time. This method can apply a range of forces from tens to hundreds of pN, with Dao et al. achieving forces as high as 600 pN [29]. Some downsides to this method are the ability to only examine one cell at a time and the possibility of the laser heating the cell. Lim et al. circumvented this by using larger beads so that the laser was not as close in proximity thereby limiting the possibility of heating [51]. Another concern is the price of this type of equipment, especially considering it requires micromanipulation. Overall, this is a useful technique as the applied force is easily controlled and maintained as long as desired, whereas in microfluidic devices the applied extensional stresses are estimated using computational methods and duration of the stress application is not controlled well.

## 3. Cell Deformability and Extensional Stresses

Cell deformability is a vital part of normal physiologic function and can give insights about different pathologies. Cells respond to mechanical forces with activation of biochemical processes, and in how they change shape under the action of the forces. How a biological cell, with its particular rheological character, will deform depends on its morphology, on its state (e.g., activated or not), and on the nature of the stresses imposed in a particular situation. Measurement of cell deformability can be valuable to determining the health of a cell and diagnosing a disease or understanding the causes of symptoms of a disease at the cellular level. For example, large numbers of cells from a patient can be examined by deformability cytometry for their mechanical phenotype in a high throughput crossflow system [52]. The method has proved effective in discerning cell pathologies from the deformabilities of cells like leukocytes and malignant cells in pleural effusions which led to accurate prediction of the disease state in patients with cancer and immune activation [53]. 

By far, the rheology of the red blood cell, in both normal and diseased states, has been most studied. With diameters of 7–8 µm, the red blood cell must be sufficiently flexible to traverse 3–5 µm capillaries and carry out its vital function of oxygen delivery to tissues. Cell deformability plays an important role in determining the lifespan of the cell in circulation as it must squeeze through slits between stress fibers in the venous sinus of the spleen [54]. As red blood cells age, they become stiff, thus making travel through the spleen difficult. The challenge of slow flow of a stiff erythrocyte in the spleen facilitates the removal by phagocytosis of an older, opsonized cell [55,56]. Cell surface-to-volume ratio, cytoplasm viscosity, and mechanical properties of the membrane all contribute to determining the rheology of red cells [57]. Abnormalities of these properties affect the mechanical character with implications for diseases like sickle cell anemia, hereditary spherocytosis, and malaria [58,59,60]. In addition, exposure of cells to supraphysiologic hemodynamic forces in prosthetic heart valves and extracorporeal circulation devices adversely affects deformability [21,61,62,63]. The result of altered rheology can be reduced red cell lifespans, anemia, an enlarged spleen and even painful episodes for individuals with sickle cell anemia.

Many methods have been used to assess deformability [38,64], but observation of cell geometry in laminar and extensional flows has been a common method. The deformability index (DI) or elongation index (EI) has been defined as (L_1_ − L_2_)/(L_1_ + L_2_) where L_1_ and L_2_ are the observed major and minor axes of the cell under flow taken from 2D microscopy images and thereby dependent on the projected area of the cells. The 2D nature of these images require the assumption of the shape of the deformed cell, such as a prolate ellipsoid [65]. This measure of cell deformability was originally developed for shearing flow of viscosity-enhanced, washed cell suspensions. Cell dimensions were determined from images obtained with interference contrast microscopy in a rheoscope [66] and by diffracted laser light in the ektacytometer [57,67]. Others have characterized deformation by the aspect ratio of the deformed cell. The advantage of DI is that it makes some accommodation to variability in cell size.

In an early study of erythrocyte deformability with an extensional flow, McGraw employed suction from two aligned, opposing 50 µm diameter micropipettes [68]. The aspect ratio for this axisymmetric elongational deformation was reported, rather than DI, to be 2.3 at a bulk stress of 3.5 dyn/cm^2^ and increasing to a maximum value of 4.8 at 5.2 dyn/cm^2^. The maximum aspect ratio of 5.3 compared favorably to 4.89 calculated for a prolate ellipsoid with the same surface area (135 μm^2^) and volume (90 μm^3^) as a human RBC [69,70,71]. The prolate ellipsoid also yields a high value of 0.68 for DI. The membrane shear elastic modulus for the RBC was found to be 0.006 dyn/cm in agreement with a value reported from micropipette experiments [72].

**Figure 4:**
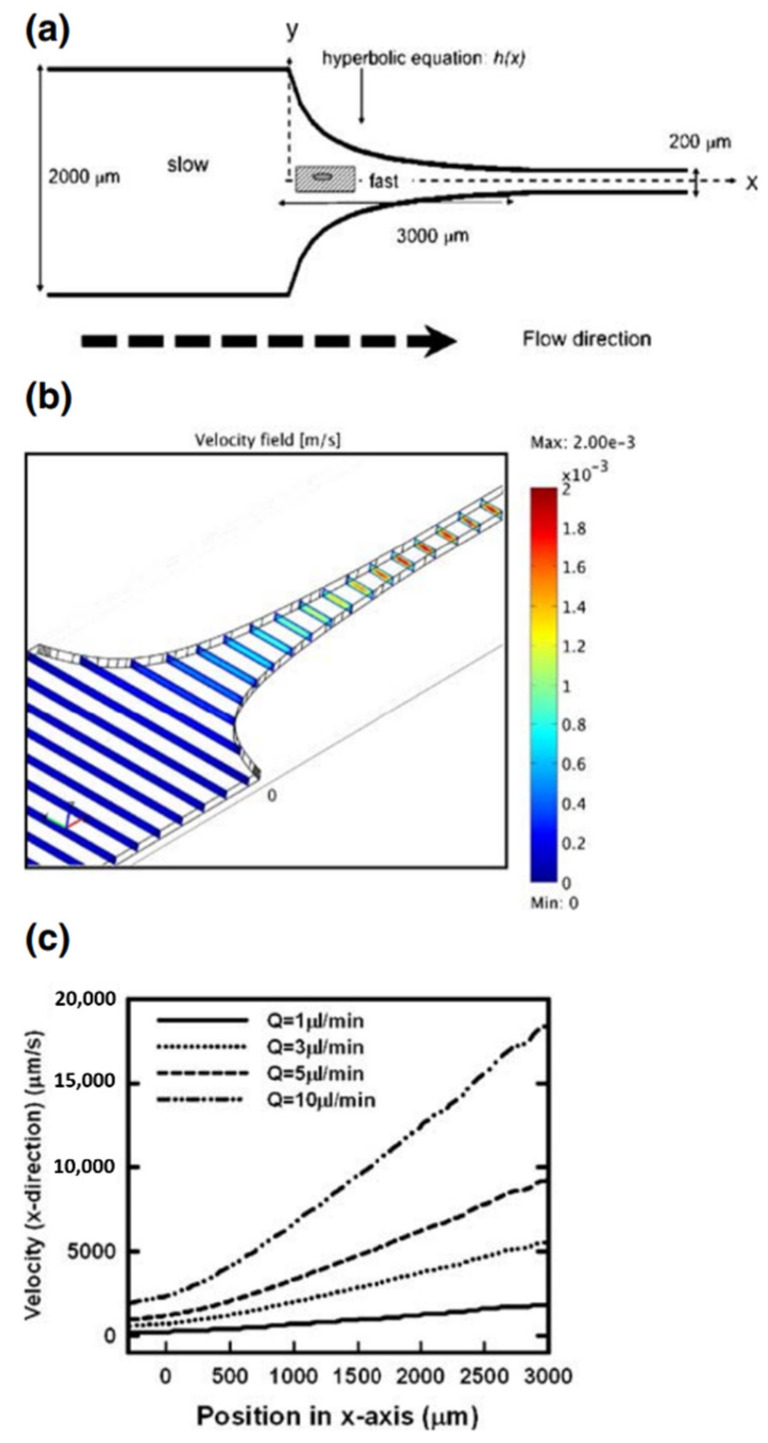
Hyperbolic channel of Lee et al. (**a**) Design-channel depth: 75 μm; cells deform in the converging section; (**b**) velocity field for Q = 1 μL/min; (**c**) centerline velocity; slope suggests $\dot{\varepsilon }$ (adapted from Springer Nature; Biomedical Microdevices, “Extensional flow-based assessment of red blood cell deformability using hyperbolic converging microchannel”, S.S. Lee et al. © 2009 [31]).

**Figure 5:**
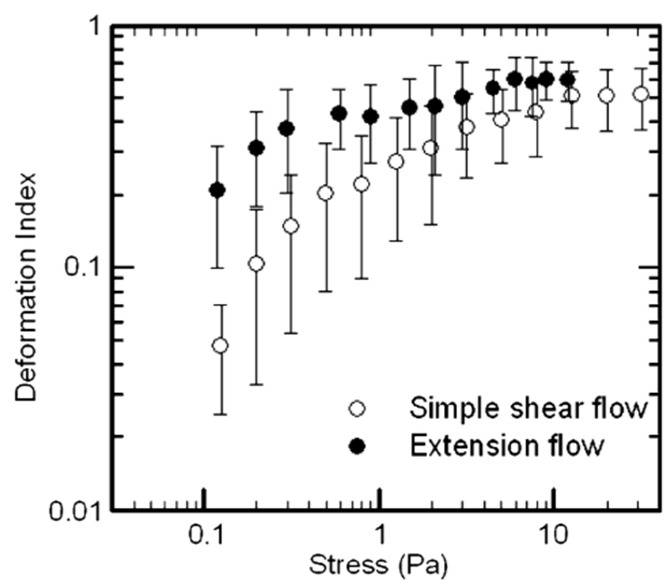
DI for red cells in shear and extensional flows (adapted from Springer Nature; Biomedical Microdevices, “Extensional flow-based assessment of red blood cell deformability using hyperbolic converging microchannel”, S.S. Lee et al. © 2009 [31]).

Several investigators have used microfluidic channels with a hyperbolic constriction to investigate the behavior of red cells in elongational flow. At a set volumetric flow rate, hyperbolic geometry produces a constant deformation rate and constant stress environment along the length of the channel for determination of deformability. The microfluidics channel used by Lee et al. is illustrated in Figure 4, along with velocity profiles for a range of volumetric flow rates [31]. They examined red cells from the rabbit in a phosphate-buffered saline with 6.8 and 12 wt% polyvinylpyrrolidone (PVP) to compare the deformation index for shear and extensional flow. PVP was added to increase the viscosity and stresses on the cell. Extensional viscosities of η_e_ = 0.093 and 0.89 Pa s were calculated from the experimental shear viscosities with Trouton’s relationship [13]. Cell images were captured with a high speed camera along or near the centerline since shear contributes to stress elsewhere. The stress and deformation index increased with the elongation rate $\dot{\varepsilon }$ as they did shear rate. Results for DI indicated much greater sensitivity of the cells to elongational flow compared to shear stress (Figure 5). At 3.0 Pa, DI of the erythrocyte was 0.51 for extensional stress compared to 0.29 for shear with respective maxima of 0.60 ± 0.106 at 9.0 Pa and 0.55 at 20 Pa. No breakup of cells was observed though the maximum stress was limited due to the experimental problem of leakage at higher flow rates. 

Faghih and Sharp constructed a system similar to Lee et al.’s channel that was able to apply higher stress levels. They were able to look at human erythrocytes in shear and elongational flow fields at stresses as high as 100 Pa [25]. The extension rate $\dot{\varepsilon }$ for their microfluidics channel was calculated from Equation (2).

Their results corroborated the key finding of Lee et al. that red cells experienced greater deformation from elongation stress as shown by larger aspect ratios for elongational flow at comparable levels of stress in a shearing flow [31]. A maximum aspect ratio of 5.3 was observed. Faghih and Sharp concluded that red cells are more sensitive to elongational stress than shear stress by an order of magnitude. While it has not been studied, the greater deformation from elongational stress as opposed to shear stress of the same magnitude suggests that elongational stresses would inflict greater damage to cells as well. Examining human RBC deformation in a hyperbolic microfluidic device, Rodrigues et al. reported a maximum observed value for DI of 0.44 [35] while Yaginuma et al. obtained a maximum DI of 0.35 in their hyperbolic channel system [30]. This latter value included a correction in the calculation of DI for displacement during exposure time for the primary axis of the cell for the video microscopy system. In addition to the correction, disagreement in reported maximum values can be attributed to species differences in the red cell, applied extension rate, and possibly an effect of viscosity [25]. In addition to studying healthy erythrocytes, hydrodynamic stretching in a hyperbolic channel has been used to examine cell pathologies. Reduced deformability, measured by the deformation ratio (length of the major axis/length of the minor axis), was observed for red cells of patients with end-stage kidney disease (ESKD) [32]. Compared to healthy controls, values were 8% less for ESKD patients without diabetes and 14% for those with diabetes. 

Mancusco and Ristenpart reported a DI value of 0.54 for human RBCs [26] using a converging microchannel with a tapered constriction and hence a non-constant deformation rate $\dot{\varepsilon }$. Applying the classic Kelvin-Voigt and Skalak constitutive equations for mechanical response, they found a high strain rate apparent membrane elastic modulus of 60 µN/m at a strain rate of 1000 s^−1^, an order of magnitude larger than reported values for low strain rates. Yen et al. utilized tapered and abrupt constrictions to measure hemolysis of porcine RBCs [10]. While they did not directly examine DI of individual cells, they did find that hemolysis correlated better with levels of extensional stress than shear stress, further supporting that extensional forces have a greater effect on red cell deformation and potential injury. DI and hemolysis have been linked in Arora et al.’s model of RBC injury [73].

A cross-flow device was used by Cha et al. to examine red cells from rats in a viscoelastic fluid (90 cp, 6.8 wt% 360,000 MW PVP in PBS) [50]. The viscoelastic fluid acted as a means of focusing the cell trajectories and facilitated capture of cells in the stagnation zone for observation. This could be helpful for the study of cell deformability since simulations show that the entry point and orientation of the cell significantly affect the deformation of the cell [27]. Cha et al. defined the elongation index as $\left(L-{\bar{L}}_{o}\right)/{\bar{L}}_{o}$ where L is the maximum observed length and ${\bar{L}}_{o}$ is the average length of the undeformed cell. The index increased with flow rate, and thus with extensional stress, to a maximum value of 1.3. For a cell diameter of 6.5 μm, this corresponds to a deformability index of about 0.4. 

Deformability for other cell types in extensional flows has not been as widely investigated. In addition to RBCs, McGraw examined leukocytes in extensional flow at 4–80 dyn/cm^2^. Subpopulations of neutrophils showed variable aspect ratios from 1.0 to 3.3 over times frames of 30–300 ms or no deformation after 2 s. The relative stiffness of leukocytes when compared to RBCs is evident with a smaller reported maximum DI of 0.10 as opposed to a maximum DI for RBCs of 0.44 with the same system [35]. As an example of the utility of extensional flow, Piergiovanni et al. looked at deformation of HL60 and Jurkat leukemia cell lines in a crossflow unit as well as a hyperbolic channel. An increase in deformability of these cells was observed on disruption of the cytoskeleton with cytochalasin D [34]. Cha et al. demonstrated increased stiffness of mesenchymal stem cells after nutrient depletion using their viscoelastic particle focusing microfluidics device [50]. In a similar system, Bae reported a maximum DI of ~0.24 for Chinese Hamster Ovary (CHO) cells [74].

Hydrodynamic stretching of cells with extensional flows has been incorporated in high throughput mechanophenotyping units. Liang et al. demonstrated the effectiveness of their system by examining the deformability of normal, crosslinked and microfilament disrupted NIH 3T3 fibroblast cells and by discerning differences between non-tumorigenic and metastatic cell lines [75]. Deformability cytometry has been used to examine cells in pleural effusions in a variety of malignancies including breast and prostate cancers as well as leukemias and lymphomas [53]. Urbanska et al. used a cross-flow microfluidic device to successfully detect a decrease in deformability of human promyelocytic leukemia cells after osmotic shock [38]. Gosset et al. used inertial focusing to deliver cells into extensional flow to examine the deformability of leukocytes and malignant cells in pleural effusions, taken from patients with immune activation and cancer [52]. They were able to accurately predict patient disease states with a sensitivity of 91% and a specificity of 86%. Gosset also examined pluripotent stem cells and showed that deformability was an early biomarker for differentiation. 

Cell deformation by extensional flow has been found to be beneficial in applications of cell biology and cell therapy. Cell therapy often utilizes the delivery of genetic nanomaterials, such as mRNA, plasmid DNA, and proteins to a variety of cell types [76]. The classical method of accomplishing this has been to use viruses or liposomes as carriers of the material or to physically perturb cellular membranes [76]. An alternative approach was described by Hur et al. which utilized a T-junction microchannel with a cavity that creates an area of elongational recirculating flows [76]. The elongational flow stretches the cell and creates membrane discontinuities which allows effective internalization of genetic nanomaterials. This method achieved superior transfection yields compared to other common methods with high efficiency, ease of use, and low material cost. 

## 4. Cell Injury by Hydrodynamic Stress

**Figure 6:**
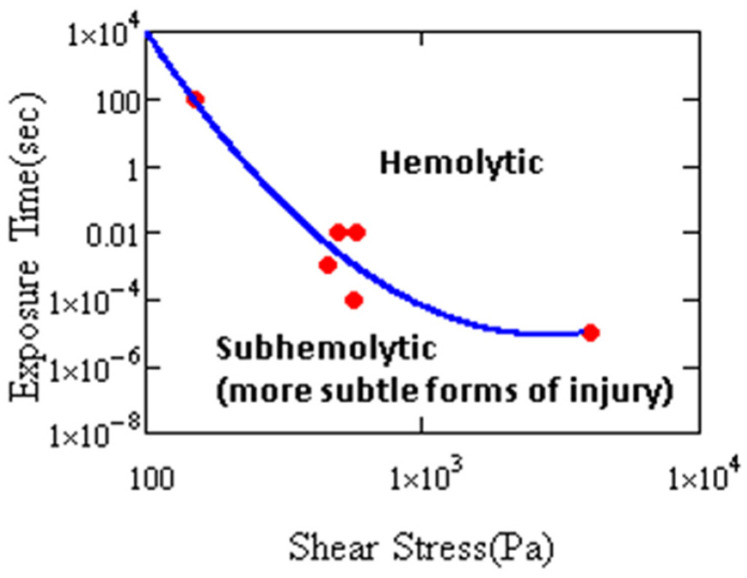
Hemolysis threshold as a function of stress magnitude and duration. Cell damage has been shown by other measures in the subhemolytic regime (after Leverett et al. [78]).

Bruises, abrasions, broken bones and cuts are macroscopic manifestations of bodily injury from mechanical forces. Trauma to tissues implies the less apparent harm at the level of cells. An appreciation for injury to discrete, individual cells grew with the development of prosthetic heart valves and artificial organs more than half a century ago as blood smears showed abnormal morphologies. Early artificial valves caused hemolysis or release of hemoglobin from RBCs as a result of the shear stresses that the blood experienced on passage. Damage as gauged by hemolysis was found to depend on exposure time and magnitude of the stress which was described in the power law equation proposed by Blackshear [77].

(5) $$HI=C\tau^{\alpha }t^{\beta }$$

where *HI* is the hemolysis index, *τ* is the magnitude of the shear stress, *t* is the exposure time and *C*, *α* and *β* are empirical constants. It should be noted that this model was purely based on effects of shear and did not account for extensional stresses. In an attempt to make a more comprehensive model, elongational forces have been incorporated by utilizing a scaler stress, such as a von Mises-like stress, that more heavily weighs the effects from shear than extensional stress. A plot of the onset of hemolysis for different devices illustrates how the threshold of hemolysis varies (Figure 6) with these flow characteristics [78]. A high stress of short duration will cause onset of hemolysis as well as a low stress of protracted duration. 

**Figure 7:**
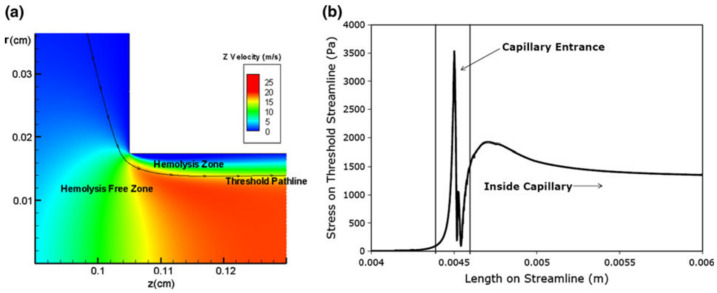
Results of flow simulation at a capillary tube entrance with contraction. (**a**) Velocity field with threshold pathline determined by hemolysis level. (**b**) Stress levels along the threshold pathline. (Reprinted by permission from Springer Nature, Annals of Biomedical Engineering, “Significance of Extensional Stresses to Red Blood Cell Lysis in a Shearing Flow” L.A. Down et al. © 2021 [82]).

Participants in the Food and Drug Administration (FDA) Critical Path Initiative sought to improve hemolysis predictions for medical device design with computer simulation. Results of experiments showed that a sudden contraction at a capillary entrance contributed to hemolysis [79] in accordance with studies years earlier by Keshaviah [80]. These findings indicate the significance of elongational stresses on damage to blood cells as can occur in aortic stenosis [71] or a rotary ventricular assist device [81]. However, the difficulty in conducting experiments with extensional flows has meant that a threshold value of stress for hemolysis remained elusive for this type of deformation and data is lacking to even confirm a dependence on exposure time and magnitude of stress. While thresholds for hemolysis were established 50 years ago, only recently has a value been determined for axisymmetric stretching of cells. Down et al. assumed an “all or none” basis for hemolysis (Figure 7) and used computational fluid dynamics to relate Keshaviah’s experimental hemolysis levels to the peak extensional stress on threshold pathlines [82]. Results yielded an estimate for the hemolysis threshold (3000 Pa) with an exposure time in the order of microseconds, while Yen et al. found a hemolysis extensional flow threshold of 1000 Pa for exposure times less than 0.060 ms [10]. 

Elongational stresses also harm blood platelets. Purvis used a constrained convergence flow chamber to expose platelets to stress at an elongational rate of 2.1 s^−1^ [83]. Cells aggregated and exhibited reduced sensitivity to ADP which was attributed to dense granule release or changes to the ADP receptor induced by stress. A potential area of future study is the effect of extensional stresses on leukocytes which are known to be sensitive to the effects of shear.

**Figure 8:**
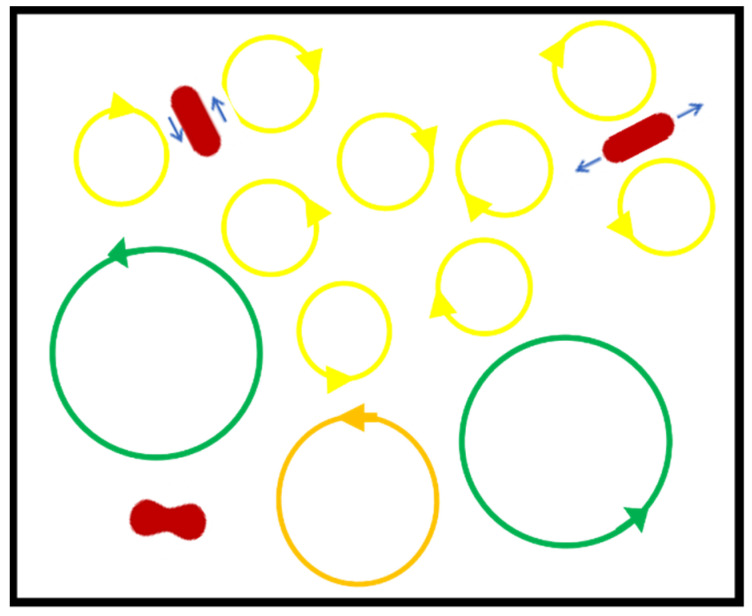
Illustration of red cell deformations in turbulent flow with Kolmogorov length scale eddies. Adjacent co-rotating eddies cause shear deformation with tank tread motion of the membrane about the cytoplasm (upper left) while counter-rotating eddies cause elongational deformation (upper right). A biconcave disc shape occurs in a region of low shear with large scale eddies.

Both shear and extensional stresses are present in turbulent flow (Figure 8) with injury by mechanical trauma possible [65,71]. The stochastic, chaotic nature of turbulence makes it hard to focus on one quantifiable turbulence characteristic to model the effects of stresses on cells and biomolecules. The Reynolds stress, defined as the time averaged product of two velocity component fluctuations in turbulent flow, has often been used but is not an effective measure of the effects of turbulence in this case. The turbulent kinetic energy and its dissipation from the flow to the RBC has been found to be a more appropriate measure of the effects of a turbulent field on the cells. The dissipation of the turbulent kinetic energy is characterized by the Kolmogorov length scale (KLS) representing the size of the smallest eddies in some region of the flow—smaller eddies than these are dissipated to thermal energy. KLS is related to energy dissipation and putting more energy into the flow results in smaller KLS. Damage to cells becomes severe when the KLS in a region of a flow field approaches the size of the cell. Aloi and Cherry found that 20 μm insect cells were harmed at KLS of similar size [84,85]. In examining 15 µm hybridoma cells, Kunas and Papoutsakis [86] determined that severe damage occurred at KLS corresponding to 13.6 µm. Similarly, Jones found that hemolysis in turbulence corresponded to Kolmogorov length scales comparable to the size to the red blood cell [87]. Such results have been supported with computational work that indicated a direct correlation between the level of hemolysis and the size and persistence of KLS in different turbulent flow fields (jets, Couette viscometers and capillary channels) [7,88,89,90]. 

The effects of mechanical trauma are also of interest to other cell types and cell pathologies. While initial work on injury to cells by fluid forces was related to medical advances, it was the emergence of the biotechnology industry that led to a focus on extensional stresses. Fragility became apparent as the bioprocess industry shifted to animal cell cultures when it was found that bacteria could not produce proteins with proper folding and glycosylation [91]. Animal cells were found to be more susceptible to harm by the shear and elongational forces present in bioreactors. Much work followed to ascertain the conditions where various cell lines experience lethal or sublethal trauma. 

The effects of hydrodynamic stress are important to the biopharmaceutical industry with worldwide production estimated to have grown to more than USD 500 billion by 2020 [91]. Cellular damage in bioreactors has been found to occur due to sparging of oxygen bubbles and turbulence from agitation. For high agitation, M. Al-Rubeai et al. described two modes of lethal destruction in a TB/C3 murine hybridoma cell line [92]. These were apoptosis and necrosis where apoptosis proceeds with blebbing, decrease in volume, and DNA condensation while cells swell and lyse with necrosis. Experiments showed that the percentage of dead cells from apoptosis increased with incubation time over the course of several days at an energy dissipation rate of 320 W m^−3^. Interestingly, G1 phase cells showed less susceptibility to injury. Zhang et al. compared TB/C3 hybridoma cells to NS1 myeloma cells in turbulent capillary flow experiments [93] and found that the myeloma cells ruptured more readily (lower energy dissipation rates). In other work, lysis of four different cell types was determined by LDH release after flow of suspensions through specially designed microfluidics channels with a structure yielding extensional and shear stresses [22]. Cells withstood maximum local energy dissipation rates as high as 10^4^–10^5^ kW/m^3^ with fragility increasing as HB-24 (mouse hybridoma), Sf-9 (insect), CHO-K1 (Chinese hamster ovary) and MCF7 (human breast carcinoma). Changes in physiological response has been observed in CHO cells at much lower energy dissipation rates than physical injury as determined by LDH [94]. However, production of monoclonal antibodies with CHO cells, the workhorse of the industry, was largely unaffected with the notable effect being on glycosylation. 

Many different parameters have been used to characterize conditions leading to cell injury by hydrodynamics including impeller speed, specific power dissipation, average wall shear stress, and KLS [95]. Both shear and extensional stresses are present in bioreactors [85] and as intrinsic properties of the flow are perhaps better suited parameters than energy dissipation rate for determining cell trauma. Using a crossflow system and a trypan blue assay of viability, Bae et al. have recently established a critical stress of ~250 Pa for injury to CHO cells by elongational stress [74].

**Figure 9:**
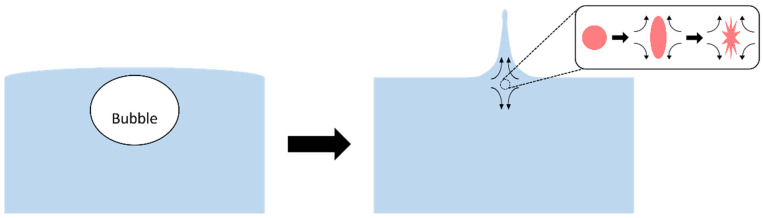
A bubble bursting at a gas–liquid interface creates upward/downward jets of fluid resulting in extensional stresses. A cell caught in the extensional flow, shown in the inset, would deform possibly to the extent of cell injury/lysis, represented by the spiked cell.

Sparging and the bursting of bubbles (Figure 9) has been considered as the main cause of cell damage in a bioreactor [96,97,98,99]. Addition of surfactant such as Pluronic F68 can mitigate the damage from the opposing jets that form as a bubble bursts at the gas–liquid interface. These create high strain rates and extensional stresses that injure cells [95,100]. Based on energy dissipation, smaller bubbles are more harmful to cells [22,101,102] with damage to insect cells being found to be inversely related to bubble size [99]. Tran conducted numerical simulations to investigate the effect of bubble diameter (0.5–6 mm) and surface tension (0.03–0.072 N/m) on the maximum extensional stress [96]. This resulted in the following equation for the maximum stress:

(6) $$\tau_{max}=9524.39\;d_{b}{}^{-1.68}\sigma^{0.46}$$

where bubble diameter *d_b_* is in mm and surface tension *σ* is in N/m and the stress is in Pa. The size of the bubble dominates a stress value with a result of 10,000 Pa obtained for a 0.5 mm bubble. 

Other researchers have investigated the harmful effects of extensional forces on cells. McQueen reported cell lysis to mouse myeloma cells (ATCC TIB 18) for turbulent flow in a capillary with extensional stresses at contraction ratios of 4–6 at the entrance [103]. On multiple passes for a wall shear stress of 180 Pa and above, cell numbers decreased, while cells that did not rupture remained viable. Similar experiments were conducted with a hybridoma cell line with lysis found as low at 80 Pa [104]. Tanzeglock et al. determined a threshold for necrosis by 60 passes through a capillary tube followed by an assay for DNA release [105]. Both Chinese Hamster Ovary cells and Human Embryonic Kidney cells lysed at an average hydrodynamic stress of 600 Pa. However, phosphatidylserine exposure and propidium iodide results indicated only 2 Pa precipitated apoptosis. Results were independent of capillary length indicating extensional rather than shear forces injured the cells.

The formation of scaffolds for tissue engineering by bioprinting is another area of technology where cell damage has been reported in the presence of elongational flow [106]. In bioprinting, cells within media such as a hydrogel are extruded to create a scaffold of the desired structure [107,108]. Bioprinting has applications in drug delivery, cancer research and organ microchip devices in addition to the formation of constructs. Ning et al. used trypan blue stain to show injury to cells (Schwann cells, HUVECs and HAT-7) after extrusion through pneumatic and screw-driven devices [106].

## 5. Extensional Stresses and Cell Disruption

The importance of extensional stresses on cells extends beyond the realm of medical and biological applications. It is also an important factor in several other areas, such as food processing, algae control in bodies of water, and biofuel production. Most of these processes utilize high pressure homogenization (HPH), which forces a sample through a small orifice at a very higher pressure, typically in the order of MPa. In other words, fluid is forced through a constriction at high pressure, inducing extensional stresses, along with shear stresses, while often turbulent flow and cavitation might be observed [109]. One application of interest of HPH is the non-thermal inactivation of pathogens in food products. It was found that the breakup, or inactivation, of *Lactococcus lactis* was most severe using a small orifice size, high pressure, and low viscosity, and that at these conditions elongational stresses and turbulence dominate the breakup of the bacteria [109]. A similar study examining the inactivation of *Escherichia coli* and *Listeria innocua* in apple and carrot juices using HPH found that increasing pressure increased inactivation, although *Listeria innocua* required higher pressures for similar levels of inactivation [110]. The disruption of *Saccharomyces cerevisiae*, or bakers’ yeast, has also been studied and it has been found that disruption occurs in the areas where extensional stresses are prevalent [111]. HPH has also been used for microbial inactivation with success in a variety of other products such as egg whites [112], orange juice, and whole milk [113]. Additionally, HPH has been used in conjunction with mild thermal treatments and nisin (an antibacterial food preservative) to achieve larger levels of bacteria inactivation in carrot juice than by HPH alone, while utilizing lower overall pressures making the process more energy efficient [114]. The use of HPH alone or in conjunction with other microbial inactivation techniques shows promise for future large-scale use, although it is limited to use only in liquid products. In addition to microbial inactivation, HPH has potential applications in other areas of food processing. Particularly, HPH has been shown to increase the stability of tomato juice by disrupting suspended pulp particles, and therefore changing the juice particle distribution size and sedimentation behavior [12]. Further applications to other products are possible where a uniform consistency and decreased sedimentation are needed. Disruption of cells by applying extensional stresses found in HPH is also beneficial for harvesting intracellular products. For example, HPH is one method of disrupting algal cells so that their intracellular macromolecules can be harvested and used in biofuel production [115]. Disrupting algae is also important in controlling algal bloom in lakes and ponds, which is vital to the survival of different fish species. Although it may be preferred to use alternative, non-mechanical methods to disrupt algae, HPH is a conventional, mechanical method that has been used in the past. It is clear that the effects of extensional stresses on different cells is important, and far reaching across many different fields. 

## 6. Conclusions and Future Work

Cells clearly respond to fluid forces in ways that can be used to determine their physical character or initiate a physiological or pathological response. While many studies have investigated the effects of shear stress, there is relatively little work on extensional stresses even though cells or at least some cell types are more sensitive to elongational deformation. In particular, more work is needed on sublethal responses and injury to extensional stresses. The development and widespread use of microfluidic systems has greatly increased the ease with which these hydrodynamic forces can be investigated or applied at the cellular level. Whether a cell is healthy, activated or stimulated, it can be probed with microfluidic devices. How cells respond to and how they are harmed by extensional stresses provide important research opportunities in fundamental and applied cell biology.

## Figures and Tables

**Table 1:** Summary of experimental methods used for creating elongational flow.

Method	Pros	Cons	Key Results
Hyperbolic Microfluidic Devices	Constant elongational rate Magnitude of extensional rate easily controlled High throughput method	Limited region of pure extensional flow Leaking at high flow rates and pressures Trajectory focusing needed	Flow instabilities at high Re and viscoelasticity [24] Achievable extensional stress of 100 Pa [25]
Abrupt/Tapered Constriction Microfluidic Devices	Easily manufactured Magnitude of extensional rate easily controlled High throughput method	Limited region of pure extensional flow Leaking at high flow rates and pressures Trajectory focusing needed	Magnitude of extensional stress higher for abrupt constrictions vs. tapered [10] Flow instabilities at high Re and viscoelasticity Achievable extensional rate of 2000 s^−1^ [26]
Cross-Flow Microfluidic Devices	Cells trapped in stagnation point are convenient to observe Bounded flow increases flow stability Magnitude of extensional rate easily controlled	One cell at a time in stagnation zone Not true planar flow Limited region of pure extensional flow Trajectory focusing needed	Flow instabilities at high Re and viscoelasticity Three possible deformation modes [27] Achievable extensional rate of 142 s^−1^ [28]
Optical Tweezers	Precise control over applied forces Wide range of forces Longer durations of applied forces	Not a high throughput method Potential of heating cell Expensive equipment	Achievable forces of 600 pN [29]
